# Disparities in Antiemetic Prophylaxis Care Processes Predicted by Patient Neighborhood: Retrospective Cohort and Geospatial Analysis

**DOI:** 10.2196/69133

**Published:** 2026-02-24

**Authors:** Jiuying Han, Neng Wan, Cameron K Jacobson, Nathan L Pace, Cade K Kartchner, Alexander Hohl, Robert B Schonberger, Douglas A Colquhoun, Richard P Dutton, Michael Andreae, John F Pearson

**Affiliations:** 1 School of Environment, Society and Sustainability, University of Utah, 260 South Central Campus Drive, Room 4625, Salt Lake City, UT, 84112, United States, 1 801-646-5989; 2 Department of Anesthesiology, Perioperative and Pain Medicine, Spencer Fox Eccles School of Medicine, University of Utah, Salt Lake City, UT, United States; 3 Department of Anesthesiology, Yale School of Medicine, New Haven, CT, United States; 4 Department of Anesthesiology, University of Michigan Medical School, Ann Arbor, MI, United States; 5 US Anesthesia Partners, Dallas, TX, United States; 6 Global Change and Sustainability Center, University of Utah, Salt Lake City, UT, United States; 7 Department of Anesthesiology, Perioperative and Pain Medicine, School of Medicine, Stanford University, 300 Pasteur DriveStanford, CA, 94305-5640, United States, 1-650-723-6412

**Keywords:** anesthesia, postoperative, geospatial analysis, database, informatics, GIS, geographic information system

## Abstract

**Background:**

Social determinants of health continue to drive persistent disparities in perioperative care. Our team has previously demonstrated racial and socioeconomic disparities in perioperative processes, notably in the administration of antiemetic prophylaxis, in several large perioperative registries. Given how neighborhoods are socially segregated in the United States, we examined geospatial clustering of perioperative antiemetic disparities.

**Objective:**

The study aimed to determine whether disparities in perioperative antiemetic prophylaxis exhibit geographic clustering based on neighborhood-level disadvantage and whether patients from disadvantaged communities are more likely to be undertreated after adjusting for individual postoperative nausea and vomiting risk.

**Methods:**

We conducted a retrospective cohort study of anesthetic records from the University of Utah Hospital involving 19,477 patients who met the inclusion criteria. We geocoded patient home addresses and combined them with the census block group–level neighborhood disadvantage, a composite index from the National Neighborhood Data Archive. We stratified our patients by antiemetic risk score and calculated the number of antiemetic interventions. We used Poisson spatial scan statistics, implemented in SaTScan (Information Management Services, Inc), to detect geographic clusters of undertreatment.

**Results:**

We identified 1 significant cluster (*P*<.001) of undertreated perioperative antiemetic prophylaxis cases. The relative risk of the whole cluster was 1.44, implying that patients within the cluster were 1.44 times more likely to receive fewer antiemetics after controlling for antiemetic risk. Patients from more disadvantaged neighborhoods were more likely to receive below-median antiemetic prophylaxis after controlling for risk.

**Conclusions:**

To our knowledge, this is the first geospatial cluster analysis of perioperative process disparities; we leveraged innovative geostatistical methods and identified a spatially defined, geographic cluster of patients whose home address census-tract level neighborhood deprivation index predicted disparities in risk-adjusted antiemetic prophylaxis.

## Introduction

### Background

Social determinants of health (SDOH) continue to drive disconcerting health care disparities [1-4]. SDOH are defined as “the societal circumstances in which we are born and grow up, learn and mature, and work and age,” and also includes race, ethnicity, education, wealth, insurance coverage, and health literacy; they impact equitable perioperative processes and outcomes as fundamental causes of disease [1,3-5]. Disparities can concern access to care, care processes*,* and health care outcomes. We focus on care processes, as the means to intervene [2,3,6]. To precisely target the underlying causes and to create, test, and implement specific countermeasures, we need to define the exact granular mechanisms leading to disparities in processes [4,5,7-10].

### Health Care Disparities in Anesthesiology and Perioperative Medicine

Disparities in care processes lead to subpar health outcomes in minoritized, marginalized, and migrant populations; differences in care processes based on race and ethnicity or socioeconomic status rather than patient risk factors and comorbidities have been a longstanding and troubling aspect of care delivery in the United States [1,3]. In outpatient settings, racial and ethnic disparities impact care for diabetes, asthma, and heart disease [11,12], as well as veterans’ care. For inpatients, race-based process-of-care surgical disparities [13] have been described, especially within neonatal and obstetric care [7,13-15]. Macario et al [16] and our team previously demonstrated process variability in anesthesiology, possibly tied to unconscious bias and negative stereotypes [2,5], as well as disparities in access to chronic pain treatment [10]. We previously demonstrated that individual clinicians provide fewer antiemetics to people who identify as Black, to those with lower health insurance, and to patients living in zip codes with lower median income [2,5]. One hypothesis is that this arises from the clinicians’ prejudice towards their patients [17]: clinicians (sub)consciously detect the social status of their patients in their interaction, and therefore become less likely to diligently elicit for, document, and treat risk factors of postoperative nausea and vomiting (PONV) [2,5]. We discussed possible concrete drivers leading to PONV disparities elsewhere [4]. Given how neighborhoods are socially segregated in the United States, the patient’s home address can provide a proxy for socioeconomic status and race [5,9]. This motivates our more granular geospatial analysis of perioperative process disparities in anesthesia care, [9]: Our primary question is whether we can identify clusters of neighborhoods with elevated social deprivation scores whose inhabitants receive less antiemetic prophylaxis after controlling for PONV risk [2,18].

### Geospatial Analysis to Investigate Health Care Disparities

**Figure 1.**
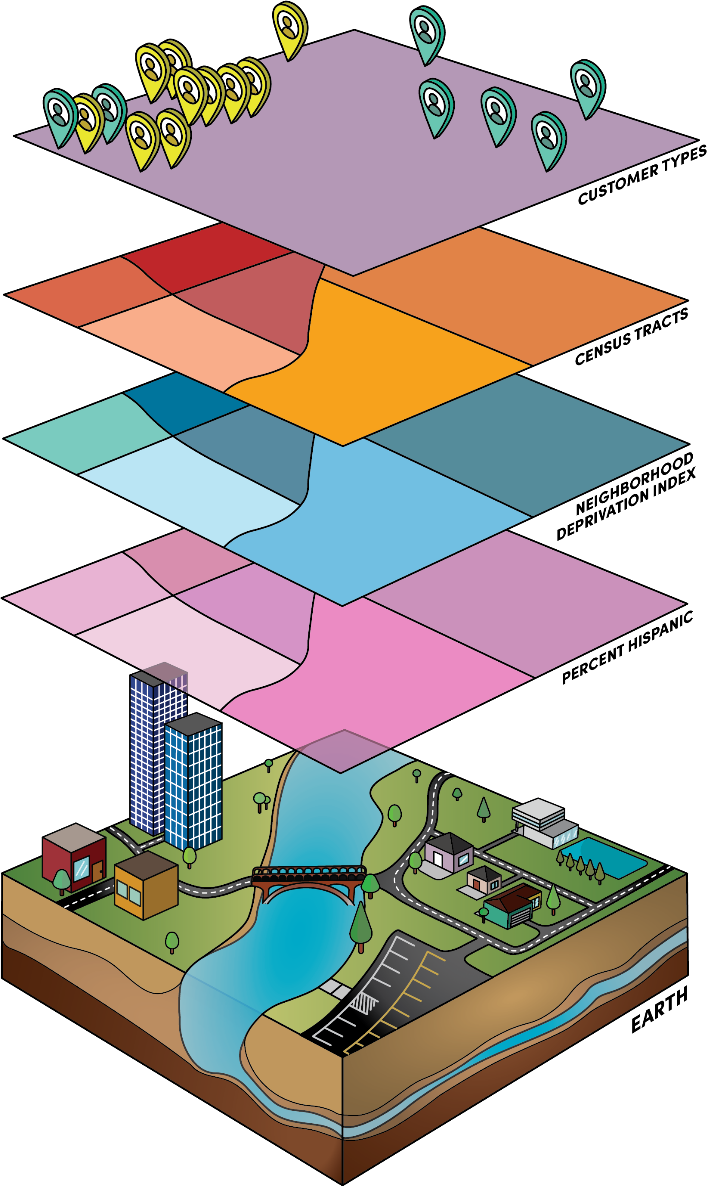
Geographic information systems are able to integrate patient home addresses, census-tract demographics, neighborhood deprivation index, and percent Hispanic to create a comprehensive view of social determinants of health.

The use of geospatial analysis to understand social and environmental influences on perioperative outcomes is a promising emerging science, with studies suggesting zip code may predict health outcomes better than a patient’s DNA [2,10]. Figure 1 illustrates the various data that can be leveraged for geospatial analysis (eg, spatial accessibility, air quality, and neighborhood deprivation). We discussed the promise and pitfalls of geospatial analysis for health systems and disparity research and the pertinent concepts previously [9]. While more mainstream in public health, very few studies have applied these innovative techniques to perioperative disparities research, especially in conjunction with large-scale perioperative registry data, such as the Multicenter Perioperative Outcomes Group (MPOG), a leading US perioperative electronic health record registry [18,19]. The integration with census-tract and block group level social determinants of health derived from publicly available databases (eg, the National Neighborhood Data Archive) further augments the power of granular electronic health registries [9]. Not only is geographically informed health care disparity research ideally suited to studying the impact of patients’ SDOH on their perioperative trajectory, but the information can also be leveraged to provide individual clinicians, teams, and institutions with actionable insights on how to improve care processes [4,7,9]. While northern Utah and the Salt Lake region have a reputation for homogeneity, the National Equity Atlas ranks Salt Lake County at 208 out of 430 US counties ranked, with a diversity index of 0.95 (diversity index is a measure of racial or ethnic diversity, with a maximum score of 1.95 if all ethnic groups are equally represented), while the United States as a whole ranges from 0.22 to 1.58, making our study population comparable to a broad swath of midsize US counties [20].

Our innovation was to apply a novel geostatistical method (Poisson spatial scan statistic) [21] to identify neighborhood-level clusters of systematic under-treatment after adjusting for PONV risk. As this methodology represents an alternative statistical approach to classical statistics, we provided Table S1 in Multimedia Appendix 1 to aid readers in interpretation of our results. We chose to use geospatial clustering, widely used in disease and crime surveillance [22,23], to explore its use of this technique in the service of health care equity. We hypothesized that patients living in neighborhoods with higher levels of social disadvantage are more likely to receive fewer perioperative antiemetics after adjusting for antiemetic risk factors, and that such neighborhoods would be found to cluster geographically [2,18,24].

## Methods

### Overview

We performed a retrospective cohort analysis of the University of Utah local MPOG electronic health registry (supplemented with University of Utah Health Epic database), enriched with geocoded SDOH for the year 2021 [25]. We adhered to the Strengthening the Reporting of Observational Studies in Epidemiology (STROBE) statement and principles (Checklist 1) [26]. All authors approved the statistical analysis plan before analyses began. We describe patient demographic characteristics, PONV risk, and perioperative antiemetic prophylaxis in Table 1.

Table 1 details characteristics of our population before and after applying exclusion criteria and filtering of data to the Wasatch Front for the purpose of our geographic analysis. Characteristics of the final study set with home addresses in the target area are listed in the final cohort data. It should be noted that the “unknown” category is unable to be disaggregated due to limitations in the underlying dataset.

Most patients in the final cohort had a median age of 48 (IQR 63-34) years and received a general anesthetic of more than 1-hour duration, including the administration of opioid medication. Their risk classification according to the American Society of Anesthesiologists (ASA) was mostly 1-3. Only 10% reported a history of PONV, and most were nonsmokers. For a median of 4 PONV risk factors, they received on average about 2 prophylactic antiemetic interventions.

The focus on the Wasatch Front area shrunk the total number of patients from 51,809 to 19,477 in the final cohort analyzed and increased the relative proportion of non-White patients (typically clustered in urban areas in Utah); the prevalence of risk factors and the number of interventions also changed. Percentages have been rounded to the nearest 10th place with some factors being described by the mean, SD, median, and IQR of the data represented.

**Table 1.** Demographic and clinical characteristics of the initial and final study cohorts.

	Original cohort		Final cohort	
Race, n (%)				
Non-White	4817 (9.3)		4254 (21.8)	
White	41,189 (79.5)		15,223 (78.2)	
Unknown	5803 (11.2)		0 (0)	
Age				
Mean (SD)	50.73 (19.68)		48.695 (17.55)	
Median (IQR)	52 (34-67)		48 (34-63)	
Sex, n (%)				
Male	22,923 (44.2)		8563 (44)	
Female	28,871 (55.7)		10,908 (56.0)	
Smoking status, n (%)				
Nonsmoker	51,112 (98.7)		19,208 (98.6)	
Anesthesia duration (>1 h), n (%)	16,270 (31.4)		7838 (40.2)	
History of PONV^a^ or motion sickness, n (%)	4608 (8.9)		2159 (11.1)	
Opioids used for postoperative pain, n (%)	45,556 (87.9)		19,176 (98.5)	
ASA PS^b^ classification, n (%)				
ASA^c^ class 1	4670 (9.0)		2411 (12.4)	
ASA class 2	20,984 (40.5)		9157 (47.0)	
ASA class 3	21,991 (42.4)		7222 (37.1)	
ASA class 4	3669 (7.1)		685 (3.5)	
ASA class 5	440 (0.8)		0 (0)	
ASA class 6	21 (0)		0 (0)	
Type of anesthesia, n (%)				
Deep sedation	21 (0)		0 (0)	
General	36,614 (70.7)		18,491 (94.9)	
MAC^d^	9814 (18.9)		0 (0)	
Regional	4499 (8.7)		986 (5.1)	
Number of risk factors				
Mean (SD)	3.46 (1.16)		3.79 (1.05)	
Median (IQR)	3 (3-4)		4 (3-4)	
Number of interventions				
Mean (SD)	1.88 (1.13)		2.49 (0.91)	
Median (IQR)	2 (1-3)		2 (2-3)	

a PONV: postoperative nausea and vomiting.

b ASA PS: American Society of Anesthesiologists Physical Status Classification.

c ASA: American Society of Anesthesiologists.

d MAC: Monitored Anesthesia Care

### Ethical Considerations

This study was considered exempt on February 9, 2023, by the institutional review board at the University of Utah (reference: 00142167). All patients at the University of Utah agreed to secondary data use as part of their consent to care, and therefore, no additional informed consent was required for this retrospective study. All data were stored on secure servers at the University of Utah consistent with university policy, and no data were transmitted outside the University firewall for geocoding, as this was done using locally installed geocoding servers. No patients were compensated for this study and no identifiable data were included in this paper.

### Geocoding SDOH

Patient home addresses were obtained from Epic and then geocoded to latitude and longitude with ArcGIS (ESRI, Inc) [9,27] and spatially matched to census block group (CBG), the boundaries of which were obtained from US Census TIGER/Line (Topologically Integrated Geographic Encoding and Referencing System) 2020 CBG boundaries layer. Any incomplete address information was excluded. We extracted neighborhood disadvantage (ND), a composite index of neighborhood deprivation, from the National Neighborhood Data Archive, a publicly available database, and used this as our independent variable [28]. ND is an average of 5 US Census indicators, including proportion of the following: non-Hispanic Black, female-headed families, households with public assistance or on food stamps, income below the federal poverty level, and unemployment, all derived from the US Census American Community Survey 5-year estimates from 2016 to 2020. ND is an average of these proportions; therefore, as this value increases, the relative disadvantage or social stress increases.

### Inclusion and Exclusion Criteria

Following the MPOG Anesthesiology Performance Improvement and Reporting Exchange (ASPIRE) quality metric PONV05 Inclusion Criteria [29], and using information obtained from the Utah local Epic and MPOG instances, we excluded cases with ASA Physical Status Classification of 5 or 6, aged <18 years, ICU transfers, and cases performed without general anesthesia (including obstetrics, ECT electroconvulsive therapy, and bronchoscopy procedures). We limited our study area further to the Wasatch Front, a densely populated metropolitan area served by the University of Utah hospital in Salt Lake City, Utah, as this better enables geospatial clustering analysis and is more generalizable to urban areas in the United States.

### Risk-Adjusted Antiemetic Prophylaxis

The novel geostatistical method (Poisson spatial scan statistic) required a characterization of patients as receiving relatively more (+1), similar (0), or fewer (−1) antiemetic interventions compared to their peers with similar PONV risk factors, leading us to define a 3-level ordinal variable of risk-adjusted antiemetic prophylaxis (RAAP), similar to the MPOG PONV-05 quality metric; RAAP is discussed in the later sections.

#### Antiemetic Interventions

The response variable was the administration of appropriate numbers of antiemetic prophylactic interventions. We counted up to 6 antiemetic interventions (0‐1, 2, 3, 4, 5, and 6+) [4,24]. These interventions included the number of and class of antiemetic prophylaxis (1 point per each), and the use of total intravenous anesthesia, as the presence of a propofol infusion, as defined in PONV05, a performance metric developed by MPOG [25].

#### PONV Risk

Risk factors for PONV likely mediate RAAP; as they could be unevenly distributed among different racially, ethnically, and geographically defined groups; PONV risk factors could confound the analysis of equitable antiemetic administration [2,4,30]. All PONV risk factors were extracted from our local MPOG instance. We categorized the PONV risk score into 6 ordinal levels (0‐1, 2, 3, 4, 5, and 6+), following the PONV05 standard of MPOG [29]. These PONV risks are defined by PONV05 and follow widely accepted guidelines including female sex (assigned at birth), history of PONV or motion sickness, nonsmoker, opioid use (either intraoperative or postoperative), duration of inhalational anesthesia greater than 1 hour, aged <50 years, and certain select procedures known to cause PONV.

#### Characterizing RAAP

First, we stratified our patients by antiemetic risk [4,24]. Within each stratum of risk, we calculated the median number of antiemetic interventions. We defined RAAP to describe above median, median, and below median RAAP. Finally, we assigned the 3 levels (below median, median, and above median) RAAP contingent on the number of prophylactic antiemetic interventions received: a high RAAP for an above median number, a median RAAP for cases with an intermediate number, and a low RAAP for cases with a below median number of antiemetic prophylactic interventions compared to other cases in the same PONV risk stratum. With RAAP, we sought to *contrast* different levels of antiemetic prophylaxis *despite* similar PONV risk, ie, process disparity driven by neighborhood-level social determinants of health [4]. Hence, RAAP serves to characterize the relative intensity of antiemetic prophylaxis after controlling for risk (not as a measure of guideline adherence) [4]. This was due to guideline variability and the use of adherence to guidelines could obscure underlying disparity within our practice, which includes physicians who trained outside of the United States, which runs counter to the purpose of the investigation.

### Cluster Analysis

We used the Poisson spatial scan statistic [21,31] implemented in SaTScan (Information Management Services, Inc) [32] to detect the clusters of undertreated perioperative antiemetic prophylaxis cases in the Wasatch Front. In this study, we focused specifically on detecting clusters of low RAAP incidence, as identifying areas with undertreatment is of particular relevance for targeting systemic interventions to improve perioperative care. A cluster contains a set of neighboring regions which collectively have higher incidence (of low RAAP) rates than expected if the low RAAP cases were evenly distributed across the study area. The Poisson spatial scan statistic uses a likelihood ratio test combined with Monte Carlo simulations to determine whether any detected clusters represent a significantly higher or lower incidence than would be expected by chance. Thus, we identify clusters only when there is statistically significant evidence of an unusually high or low rate of under-treatment relative to the expected distribution under the null model.

We used univariate and multivariate logistic regressions to investigate the characteristics of the CBG in the identified low RAAP cluster. The outcome was a binary variable indicating whether the CBG belongs to the high-rate low RAAP or not. We tested previously described or suspected social determinants of health as predictors of disparities: [2,5] percentage of non-White population, percentage of population with education less than high school, percentage of population below poverty rate, percentage of population married, percentage of older people (aged >65 y), and percentage of renters. Finally, the presence of spatial autocorrelation was tested by calculating the Moran I statistic for the residuals of the multivariate model.

## Results

### Description of the Population and Study Flow Diagram

**Figure 2.**
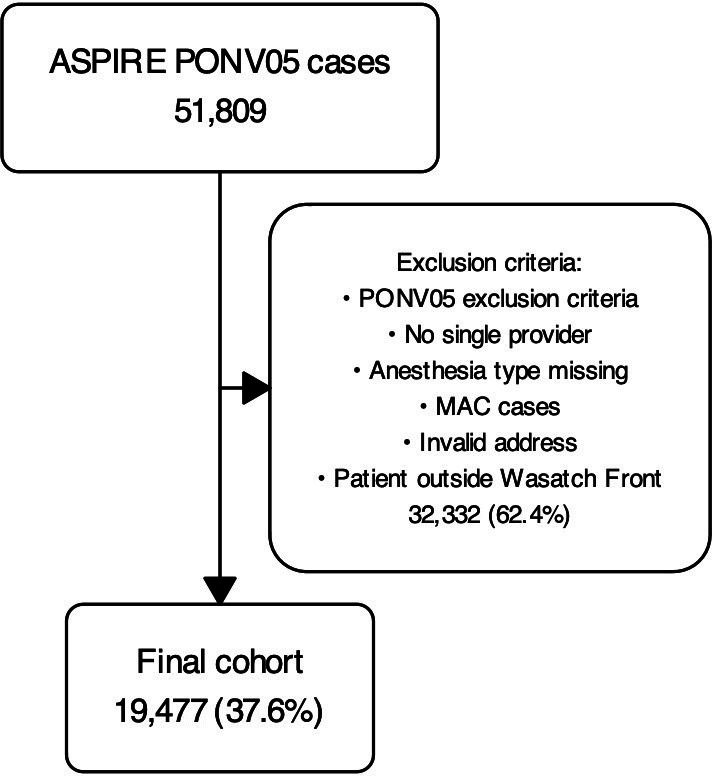
Cohort selection flow diagram of 51,809 cases with exclusions and final analytic cohort of 19,477 cases. ASPIRE: Anesthesiology Performance Improvement and Reporting Exchange; MAC: monitored anesthesia care (excluded); MPOG: Multicenter Perioperative Outcomes Group; PONV: postoperative nausea and vomiting.

A total of 51,809 anesthetic case records of patients undergoing surgery at the University of Utah were extracted from the Utah MPOG 2021 dataset. After excluding cases with missing data, a final dataset of 19,477 cases was analyzed in the cluster analysis, as detailed in Figure 2 with missing data noted therein. Table 1 lists detailed characteristics of the original cohort as well as the final analysis cohort after applying our exclusions criteria.

The median age in the final cohort was 48 (IQR 34‐63) years; 94.9% (18,491/19,477) of patients underwent a general anesthetic, often lasting longer than 1 hour (7838/19,477, 40.2%); the administration of opioid medication was almost always part of the anesthetic plan (19,176/19,477, 98.5%). ASA class was mostly 1‐3 (2411/19,477, 12.4%; 9157/19,477, 47.0%; and 7222/19,477, 37.1%, respectively). Approximately one-tenth of patients reported a history of PONV. Most patients were nonsmokers (19,208/19,477, 98.6%). The median number of PONV risk factors was 4 (IQR 3‐4), and patients received a median of 2 (IQR 2‐3) prophylactic antiemetic interventions.

The final cohort had approximately the same percentage of White patients (41,189/51,809, 79.5% original vs 14,223/19,477, 78.2% final; standardized mean difference [SMD] 0.03), similar sex distribution (28,871/51,809, 55.7% female original vs 10,908/19,477, female 56.0% final; SMD 0.01) and similar smoking status (51,112/51,809, 98.7% nonsmoking original vs 19,208/19,477, 98.6% nonsmoking final; SMD 0.00) while the final cohort had slightly more risk factors (3.46 vs 3.79; SMD 0.29), interventions (1.88 vs 2.44, SMD 0.55), and higher prevalence of a lower ASA (ASA1-2: 25,654/51,809, 50% original vs 11,568/19,477, 59% final; SMD 0.27).

### Uni- and Bivariate Analysis

In total, we found 2260 low RAAP cases, 7358 median RAAP, and 9589 high RAAP cases in our final dataset. The association between socioeconomic neighborhood-level and demographic patient factors and RAAP is presented in a bivariate tabulation (Table 2).

Table 2 demonstrates how home address and race predicted RAAP in a descriptive, bivariate comparison. Levels of RAAP are tabulated in 3 columns: below median, median, and above median, which reflect disparities. The ND scores in the rows were separated into quartiles, with the 1st quartile Q1 being the lowest score (meaning the least disadvantaged) and the 4th quartile being the highest disadvantaged score (meaning the most disadvantaged). Populations living in more disadvantaged neighborhoods (eg, Q4=ND 4th quartile) on average received fewer antiemetic interventions after adjusting for risk, with 17.1% (835/4869) of Q4 receiving low RAAP versus 14.2% (692/4870) low RAAP for Q1 (most affluent) and similarly 41.1% (2001/8850) of Q4 receiving high RAAP versus 49.1% (2390/8850) of Q1 receiving high RAAP; hence, they were relatively undertreated compared to patients living in more affluent neighborhoods (Q1). In addition, people who self-identified as White mostly received many antiemetic interventions after controlling for risk (only 933/8850, 10.5% low RAAP) compared with unknown patients who more often received fewer interventions after controlling for risk (470/2986, 15.7% low RAAP) and non-White patients (897/7641, 11.7% low RAAP).

**Table 2.** Association of neighborhood disadvantage and race with risk-adjusted antiemetic prophylaxis (RAAP).

Category	RAAP			Total, n (%)
	High RAAP, n (%)	Median RAAP, n (%)	Low RAAP, n (%)	
Quartile				
Q1	2390 (49.07)	1788 (36.71)	692 (14.21)	4870 (25)
Q2	2331 (47.87)	1858 (38.16)	680 (13.96)	4869 (25)
Q3	2128 (43.70)	1962 (40.29)	779 (16.41)	4869 (25)
Q4	2001 (41.09)	2033 (41.75)	835 (17.14)	4869 (25)
Total	8850 (45.43)	7641 (39.23)	2986 (15.33)	19,477 (100)
Race				
Non-White	5990 (78.39)	754 (9.86)	897 (11.74)^a^	7641 (39.23)
White	7097 (80.19)	820 (9.26)	933 (10.54)^b^	8850 (45.43)
Unknown	2136 (71.53)	380 (12.73)	470 (15.74)^a^	2986 (15.33)
Total	15,223 (78.15)	1954 (10.03)	2300 (11.81)	19,477 (100)

a *P*<.001

b *P*=.003

Finally, an asymptotic generalized Pearson chi-squared test was performed to compare the distribution of RAAP values between all pairwise race categories (White, non-White, and unknown). The associated *P* values are as follows: White versus non-White (*P*=.003), White versus unknown (*P*<.001), and non-White versus unknown (*P*<.001).

Patient self-identified race predicted RAAP. Living in more disadvantaged neighborhoods (eg, Q4=ND 4th quartile) was associated with receiving fewer antiemetic interventions after adjusting for risk (835/4869, 17.1% low RAAP); in other words, compared to patients living in more affluent neighborhoods (Q1=1st ND quartile with 2390/8850, 49.0% high RAAP), clinicians administered fewer antiemetic interventions, even after adjusting for risk, in cases of patients living in CBGs with higher ND scores, which represent neighborhoods with higher proportions of non-Hispanic Black, female-headed families, households with public assistance or on food stamps, income below federal poverty level, and unemployment. In addition, after controlling for PONV risk, patients who self-identified as White received more antiemetic interventions (7097/8850, 80.1% high RAAP; *P*=0.003), and rarely fewer antiemetic interventions (only 933/8850 10.5% low RAAP; *P*<.001) while non-White patients more often received fewer interventions after controlling for risk (470/2986, 15.7% low RAAP; *P*<.001), compared to White patients in similar PONV risk strata. These associations are congruent with the results of our more complex statistical models.

The bivariate analyses showed that all the variables of interest, except age, were significantly related to being inside the cluster, with the percentage of non-White, percentage of male, percentage of education less than high school, percentage of population below poverty level, and the percentage of renter being positively related to belonging to a high-rate cluster, while the percentage of married population being negatively related to belonging to a cluster.

### Geospatial Analysis

We identified 1 significant cluster (*P*<.001) of low RAAP cases in Wasatch Front (Multimedia Appendix 2), comprising 101 CBGs. The cluster was located around the geographic center of Salt Lake City, and included West Valley City, South Salt Lake, and Taylorsville. The cluster had a total of 1934 included participants residing within it, or approximately 9.93% of the study population. The relative risk (RR) of the whole cluster was 1.44 (*P*<.001), implying that the risk of receiving less (low RAAP) was 1.44 times higher for patients living *within* the cluster than for those living in other CBGs along the Wasatch Front. The CBG level RR within the cluster ranged between 0 and 3.73 (with RR>1 being a high relative risk), meaning the highest risk of low RAAP was 3.73 times higher living in CBGs within versus outside the cluster.

After adjusting for all listed covariates, we found that percentage of age >65 years in a CBG cluster became highly significantly associated with a low RAAP cluster, which would represent guidance-congruent care as age protects against PONV. However, we similarly demonstrated that the percentage of non-White people in a geographic area was also associated with low RAAP, which represents an unexplained process disparity. The larger the percentage of age >65 years and the percentage of non-White, which in West Valley City is over 42% Hispanic [33], the more likely the CBG belonged to the low RAAP cluster. In addition, the percentage of males was positively related to belonging to a low RAAP cluster, but with borderline statistical significance (again indicating guidance-congruent care, as female sex is positively associated with PONV risk). The Moran I statistic (Moran I=0.003; *P*=.79) indicated no evidence of spatial autocorrelation in the model residuals.

## Discussion

### Principal Findings

In our retrospective cohort of patients at the University of Utah local MPOG electronic health registry [25], we leveraged a novel geostatistical method to identify a spatial cluster of patients receiving less RAAP than peers in similar PONV risk strata [32]. We identified one significant cluster (*P*<.001) of low RAAP cases in the Wasatch Front (Multimedia Appendix 2), comprising 101 CBGs.

The cluster identified is an area of low RAAP cases with a high ND score (reflecting structural neighborhood features such as female-headed families, households with public assistance or on food stamps, income below the federal poverty level, and unemployment), and a relatively higher percentage of Hispanic and non-White patients. Low RAAP, defined in the methods, reflects process disparities in RAAP. Our findings are consistent with a direct association between ND and RAAP. Our results remained statistically significant in multivariate analysis, where we demonstrated that non-White individuals received lower RAAP than self-identified White participants. Uni- and bivariate tabulation of our data (Table 2) corroborated our principal geospatial analysis, while the association of male sex and older age in CBGs with low RAAP was consistent with guidance-congruent care, as both patient characteristics are associated with lower PONV risk.

### Interpretation of Principal Findings

Only individual patient-level PONV risk factors should be the guiding principle of RAAP [2,4,5,24]; neither patient race and ethnicity, nor ND should impede equitable RAAP [2,18,24]. By identifying geographic clusters of low RAAP, representing populations experiencing disparities in antiemetic prophylaxis after adjusting for risk, we visualized how geographically defined SDOH drive health care process disparities within anesthesiology [9]. Our finding that those within a disadvantaged geographic area, in this case the Hispanic-dominated West Valley region of Salt Lake County (where West Valley City has over 42% identify as Hispanic) [33], are over 40% less likely to receive RAAP is troubling, and it reinforces the use of geospatial analysis to identify populations at risk of process disparities. It should be further noted that the use of the spatial scan statistic examines individual CBGs in the context of neighboring CBGs: the ability to analyze beyond administrative boundaries can capture broader area characteristics, an advantage when examining subdivisions within a broader area of disparity.

### Significance of Geospatial Process Disparities in the Literature

Why would a patient’s home address determine how many antiemetics they receive, as we demonstrated in this geospatial analysis? Geographic analyses in perioperative medicine have been sparse [34-36], and prior work tended to focus on patient *outcomes* rather than on *process* disparities. Geographic clusters of disparities in care have been well-documented in various areas of medicine, including diabetes, asthma, heart disease, veterans care, surgical care, as well as neonatal care [7,11-15]. Our findings also build on process outcomes in pediatric anesthesiology and our own work in obstetric care pain medicine, where cancellation rates were found to be higher in communities with lower socioeconomic status [10,19,35,37,38]. Our findings also add to our prior research on disparities in RAAP driven by patient- and neighborhood-level social determinants of health (patient race, insurance, and median income in zip code) [2,5]. Our results are consistent with these prior findings but examine perioperative process disparities through a geospatial lens with granular geostatistical methods, considering proximity statistically [9,21]. In addition, compared to previous work, the cluster analysis used here offers 2 key advantages. First, zip codes, designed primarily for mail delivery, are often misaligned with meaningful health behaviors or risk boundaries and can introduce bias when used as analytic units [39,40]. Second, by applying spatial scan statistics to pinpoint geographic clusters of suboptimal perioperative care, we move beyond coarse aggregation to directly identify and quantify localized disparities. The geospatial granularity empowers public health officials to deploy interventions (eg, quality improvement initiatives, tailored community outreach, and provider education) focused precisely on those areas where care gaps are statistically significant, thus enhancing the effectiveness of efforts to improve perioperative outcomes.

### Promise of Geospatial Analysis of Disparities

The innovation of our study is to perform a geospatial analysis of perioperative process disparities, leveraging equitable RAAP as a case study [2,5]. This represents an important step forward in terms of ability to ameliorate disparities, as geospatial drivers can be reevaluated over time, for example, after mitigation measures are put in place to address disparities [9]. The benefits of exploring process differences by neighborhood were also noted in studies in Scotland and California [41]; identifying specific neighborhood clusters provided for targeted outreach to improve diabetes care.

Our analysis of local catchment areas with disparities in care *processes*, for which we are individually accountable as clinicians [5], can make for a different conversation within a department and among clinicians about potential modifications to practice patterns; these may ultimately drive many outcome disparities which some may have traditionally considered beyond our direct influence and scope of practice [7]. Outcome differences may be influenced by environmental factors (eg, pollution) while differences in risk-adjusted processes, under the primary domain of the individual anesthesia provider in the operating room, leave fewer excuses [4], though our findings are based on aggregate patterns and should not be interpreted as direct evidence of bias at the individual provider level. Despite this limitation, a focus on process provides greater insight into clinician behaviors and relationships with patients, with neighborhood structural factors potentially leading to care disparities potentially shaping the provider-patient relationship in the high-stakes and brief time of the preoperative anesthesia assessment and contributing to cumulative disadvantage. It also identifies, in our instance, an area where language barriers (due to Hispanic, Spanish-speaking population) may impede recognition of PONV risk factors and thus opens the door to potential novel avenues to improve care [4,10]. In our institution, identifying that a Hispanic, Spanish-speaking predominant neighborhood is potentially experiencing disparities is a unique way that geospatial clustering uncovers actionable, neighborhood-targeted hypotheses for intervention.

Studies from Taiwan and the United Kingdom National Health Service system show a complex interplay between care disparities and socioeconomic status of the neighborhood, demonstrating that even in systems with near-universal health care coverage, socioeconomic status can drive disparities in the delivery of care [42,43]. In contrast, other studies have suggested that neighborhood characteristics have only a modest impact on some care processes and outcomes [44], while the influence of race may overshadow neighborhood characteristics.

### Limitations and Potential Bias in Our Methods

Our study is not without its limitations. First, limiting generalizability, we confined the study population to the catchment area of the University of Utah Hospital, limited to 1 health system and 1 group of anesthesiologists. However, our prior studies around race have examined multiple hospitals in the large perioperative electronic health registries with analogous disparities identified [2,5]. Furthermore, the racial and ethnic diversity of populations varies across the United States, somewhat limiting the external validity of our study to states with lower minority representation. Low RAAP areas had low socioeconomic status and higher rates of Hispanic populations. This could result in autocorrelation and bias, as it is possible that neighborhood socioeconomic status and geographic factors were less salient than race but could not be fully separated in our model due to our small patient cohort. Expanding our approach to include areas of economic diversity among the Hispanic or other minoritized populations (eg, Texas, South Florida, and California) would further elucidate this relationship. The MPOG registry and our local dataset do not provide data on the race, sex, or ethnicity of the *clinicians* assigned to a particular anesthetic case. We were therefore unable to investigate if congruence of identity characteristics between patients and clinicians for a given case had an impact on clinician adherence with the best antiemetic practices. Furthermore, the assignment of staff to specific clinical locations could introduce bias in our results, especially if a single subgroup regularly practices at a given location; however, as the vast majority of cases take place at the main University of Utah clinical campus, the potential for this bias is minimal.

Another limitation, inherent in all geographic epidemiological retrospective studies, is the ecological fallacy [9,45]; we did not have individual self-identified race for all patients, as our local instance of MPOG and Epic provides inconsistent information on race and ethnicity, thus this is a data quality gap that constrains our interpretation. Our most reliable information is aggregate data at the neighborhood CBG level. Despite joining some individual data with group-level data, the risk of ecological bias, while mitigated, is still present. We acknowledge potential *missing data bias*, since we were only able to identify 93.1% of patients’ addresses to the level of the street point address, (which is, however, consistent with other geocoding results) [9,46]. We also acknowledge that limiting the study area to the Wasatch Front, which was done to ensure contiguous CBGs for geospatial clustering analysis, may have resulted in the exclusion of rural disparities. We also did not examine the race of clinicians or perform any analysis by clinician characteristics [4]. A few outliers could have skewed the cohort, though this is unlikely given the inclusion of over 100 anesthesiologists. More detailed strengths and weaknesses of our geospatial analysis approach are discussed in Multimedia Appendix 1.

### Conclusions

Demonstrating the use of a novel geostatistical method in a retrospective cohort of anesthesia case records from the local MPOG electronic health registry at the University of Utah, we identified a spatial cluster of patients in the West Valley area of Salt Lake City, Utah, receiving less RAAP (Multimedia Appendix 2); they also represented a population from CBGs with relatively high neighborhood deprivation. Our results are consistent with prior work that demonstrated disparities in antiemetic prophylaxis based on *patient-*level factors (eg, race) [2], but adding a *neighborhood*-level, spatial dimension to perioperative health care disparities and health systems research, with many potential applications to elucidate geographic, social, and environmental drivers of the health care process and outcome (Figure 1) [9].

## Supplementary material

10.2196/69133 Multimedia Appendix 1 Strengths and weaknesses of different spatial analysis approaches.

10.2196/69133 Multimedia Appendix 2 Map of the Wasatch Front highlighting cluster of under-treatment in risk-adjusted antiemetic prophylaxis identified by SaTScan (Information Management Services, Inc). This corresponds to an area of Salt Lake County with a high prevalence of Hispanic population.

10.2196/69133 Checklist 1 STROBE checklist.

## Abbreviations

ASA: American Society of Anesthesiologists
ASPIRE: Anesthesiology Performance Improvement and Reporting Exchange
CBG: census block group
MPOG: Multicenter Perioperative Outcomes Group
ND: neighborhood disadvantage
PONV: postoperative nausea and vomiting
RAAP: risk-adjusted antiemetic prophylaxis
RR: relative risk
SDOH: social determinants of health
SMD: standardized mean difference
STROBE: Strengthening the Reporting of Observational Studies in Epidemiology
TIGER/Line: Topologically Integrated Geographic Encoding and Referencing System
