# Supplementary figures and images for: Disparities in Antiemetic Prophylaxis Care Processes Predicted by Patient Neighborhood: Retrospective Cohort and Geospatial Analysis
Source: JMIR Public Health Surveill. 2026 Feb 24;12:e69133. doi: 10.2196/69133 (PMC12936653; doi:10.2196/69133)

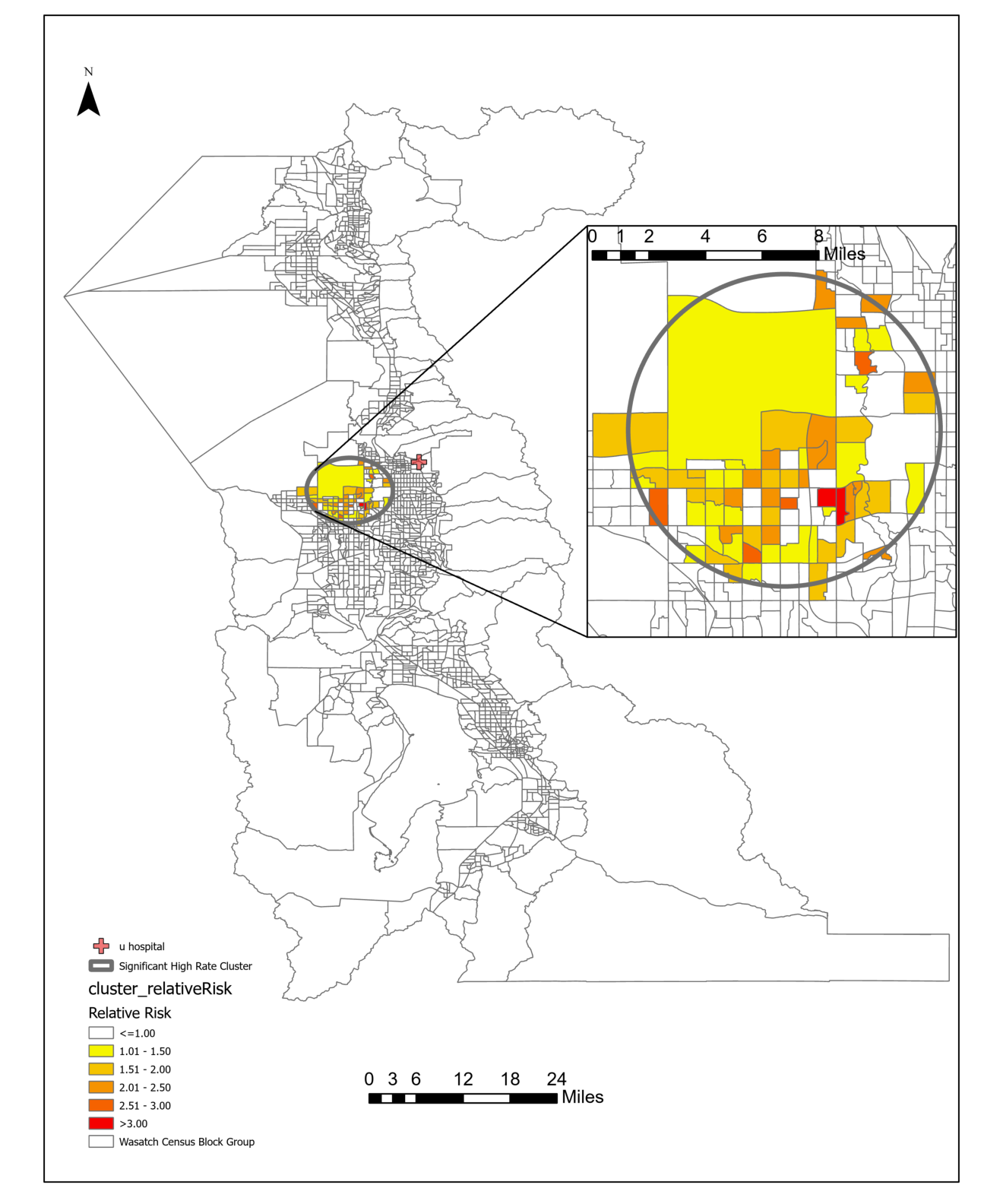

Supplement: Multimedia Appendix 2 [file publichealth-v12-e69133-s002.png]
