# Supplementary material for: Disparities in Antiemetic Prophylaxis Care Processes Predicted by Patient Neighborhood: Retrospective Cohort and Geospatial Analysis
Source: JMIR Public Health Surveill. 2026 Feb 24;12:e69133. doi: 10.2196/69133 (PMC12936653; doi:10.2196/69133)
Supplement: Checklist 1 [file publichealth-v12-e69133-s003.docx]

STROBE Statement—checklist of items that should be included in reports of observational studies

|  | Item No. | Recommendation | Page  No. | Relevant text from manuscript |
| --- | --- | --- | --- | --- |
| **Title and abstract** | 1 | (*a*) Indicate the study’s design with a commonly used term in the title or the abstract | Abstract | **Objective**: We sought to determine whether disparities in perioperative antiemetic prophylaxis exhibit geographic clustering based on neighborhood-level disadvantage, and whether patients from disadvantaged communities are more likely to be undertreated after adjusting for individual post-operative nausea and vomiting risk. |
|  |  | (*b*) Provide in the abstract an informative and balanced summary of what was done and what was found | Abstract | **Methods:** We conducted a retrospective cohort study of anesthetic records from the University of Utah Hospital. We identified one significant cluster (p < .001) of undertreated perioperative antiemetic prophylaxis cases. Patients from more disadvantaged neighborhoods were more likely to receive below median antiemetic prophylaxis after controlling for risk. |
| Introduction | | | |  |
| Background/rationale | 2 | Explain the scientific background and rationale for the investigation being reported | Introduction | Social determinants of health (SDoH) continue to drive disconcerting healthcare disparities[1-4]. We previously demonstrated that individual clinicians provide fewer anti-emetics to people who identify as Black, to those with lower health insurance, and to patients living in zip codes with lower median income[2, 5]. Given how neighborhoods are socially segregated in the US, at the patient’s home address can provide a proxy for socioeconomic status and race[5, 9]. |
| Objectives | 3 | State specific objectives, including any prespecified hypotheses | Introduction | We hypothesized that patients living in neighbourhoods with higher levels of social disadvantage are more likely to receive fewer perioperative antiemetics after adjusting for antiemetic risk factors, and that such neighbourhoods would be found to cluster geographically[2, 18, 24]. |
| Methods | | | |  |
| Study design | 4 | Present key elements of study design early in the paper | Materials and Methods | We performed a retrospective cohort analysis of the University of Utah local MPOG electronic health registry. |
| Setting | 5 | Describe the setting, locations, and relevant dates, including periods of recruitment, exposure, follow-up, and data collection | Materials and Methods | University of Utah Hospital, 2021, MPOG registry and Epic data. |
| Participants | 6 | (*a*) *Cohort study*—Give the eligibility criteria, and the sources and methods of selection of participants. Describe methods of follow-up  *Case-control study*—Give the eligibility criteria, and the sources and methods of case ascertainment and control selection. Give the rationale for the choice of cases and controls  *Cross-sectional study*—Give the eligibility criteria, and the sources and methods of selection of participants | Materials and Methods | Following the MPOG/ASPIRE quality metric PONV05 Inclusion Criteria,[29] and utilizing information obtained from the Utah local Epic and MPOG instances, we excluded cases with American Society of Anesthesiologists Physical Classification Status (ASA) of 5 or 6, age<18 years, ICU transfers, and cases performed without general anesthesia (including Obstetric, ECT electroconvulsive therapy, and bronchoscopy procedures). We limited our study area further to the Wasatch Front, a densely populated metropolitan area served by the University of Utah hospital in Salt Lake City, UT, as this better enables geospatial clustering analysis and is more generalizable to urban areas in the US. |
|  |  | (*b*) *Cohort study*—For matched studies, give matching criteria and number of exposed and unexposed  *Case-control study*—For matched studies, give matching criteria and the number of controls per case | Materials and Methods | Following the MPOG/ASPIRE quality metric PONV05 Inclusion Criteria,[29] and utilizing information obtained from the Utah local Epic and MPOG instances, we excluded cases with American Society of Anesthesiologists Physical Classification Status (ASA) of 5 or 6, age<18 years, ICU transfers, and cases performed without general anesthesia (including Obstetric, ECT electroconvulsive therapy, and bronchoscopy procedures). We limited our study area further to the Wasatch Front, a densely populated metropolitan area served by the University of Utah hospital in Salt Lake City, UT, as this better enables geospatial clustering analysis and is more generalizable to urban areas in the US. |
| Variables | 7 | Clearly define all outcomes, exposures, predictors, potential confounders, and effect modifiers. Give diagnostic criteria, if applicable | Materials and Methods | The response variable was the administration of appropriate numbers of antiemetic prophylactic interventions. We extracted “neighborhood disadvantage”(ND), a composite index of neighborhood deprivation, from the National Neighborhood Data Archive (NaNDA), a publicly available database, and used this as our independent variable.[28] Risk factors for PONV likely mediate risk adjusted antiemetic prophylaxis; as they could be unevenly distributed among different racially, ethically, and geographically-defined groups; PONV risk factors could confound the analysis of equitable antiemetic administration.[2, 4, 30] |
| Data sources/ measurement | 8* | For each variable of interest, give sources of data and details of methods of assessment (measurement). Describe comparability of assessment methods if there is more than one group | Materials and Methods | Patient home addresses were obtained from Epic and then geocoded to latitude and longitude with ArcGIS (ESRI, Inc.)[9, 27] and spatially matched to Census Block Group (CBG) the boundaries of which were obtained from US Census TIGER/Line 2020 CBG boundaries layer. We extracted “neighbourhood disadvantage”(ND), a composite index of neighborhood deprivation, from the National Neighborhood Data Archive (NaNDA), a publicly available database, and used this as our independent variable.[28] The response variable was the administration of appropriate numbers of antiemetic prophylactic interventions. |
| Bias | 9 | Describe any efforts to address potential sources of bias | Materials and Methods | Risk factors for PONV likely mediate risk adjusted antiemetic prophylaxis; as they could be unevenly distributed among different racially, ethically, and geographically-defined groups; PONV risk factors could confound the analysis of equitable antiemetic administration.[2, 4, 30] All PONV risk factors were extracted from our local MPOG instance. Finally, the presence o spatial autocorrelation was tested by calculating Moran’s I statistic for the residuals of the multivariate model. |
| Study size | 10 | Explain how the study size was arrived at | Materials and Methods | Following the MPOG/ASPIRE quality metric PONV05 Inclusion Criteria,[29] and utilizing information obtained from the Utah local Epic and MPOG instances, we excluded cases with American Society of Anesthesiologists Physical Classification Status (ASA) of 5 or 6, age<18 years, ICU transfers, and cases performed without general anesthesia (including Obstetric, ECT electroconvulsive therapy, and bronchoscopy procedures). We limited our study area further to the Wasatch Front, a densely populated metropolitan area served by the University of Utah hospital in Salt Lake City, UT, as this better enables geospatial clustering analysis and is more generalizable to urban areas in the US. We present in Figure 2, a Quorum Flow Chart, with missing data noted therein. |

Continued on next page

| Quantitative variables | 11 | Explain how quantitative variables were handled in the analyses. If applicable, describe which groupings were chosen and why |  |  |
| --- | --- | --- | --- | --- |
| Statistical methods | 12 | (*a*) Describe all statistical methods, including those used to control for confounding | Materials and Methods | We utilized Poisson Spatial Scan Statistic[21, 31] implemented in SaTScan (http://www.satscan.org/)[32] to detect the clusters of under-treated perioperative antiemetic prophylaxis cases in the Wasatch Front. The Poisson spatial scan statistic uses a likelihood ratio test combined with Monte Carlo simulations to determine whether any detected clusters represent a significantly higher or lower incidence than would be expected by chance. We used univariate and multivariate logistic regressions to investigate the characteristics of the CBG in the identified low RAAP cluster. |
|  |  | (*b*) Describe any methods used to examine subgroups and interactions | Materials and Methods | We used univariate and multivariate logistic regressions to investigate the characteristics of the CBG in the identified low RAAP cluster. We tested previously described or suspected social determinants of health as predictors of disparities:[2, 5] percentage of non-White population, percentage of population with education less than high school, percentage of population below poverty rate, percentage of population married, percentage of elderly (> 65years), and percentage of renters. Finally, the presence of spatial autocorrelation was tested by calculating Moran’s I statistic for the residuals of the multivariate model. |
|  |  | (*c*) Explain how missing data were addressed | Materials and Methods | Any incomplete address information was excluded. We present in Figure 2, a Quorum Flow Chart, with missing data noted therein. Table 1 details characteristics of our population prior and after applying exclusion criteria and filtering of data to the Wasatch Front for the purpose of our geographic analysis. |
|  |  | (*d*) *Cohort study*—If applicable, explain how loss to follow-up was addressed  *Case-control study*—If applicable, explain how matching of cases and controls was addressed  *Cross-sectional study*—If applicable, describe analytical methods taking account of sampling strategy |  |  |
|  |  | (*e*) Describe any sensitivity analyses | Materials and Methods | First, we stratified our patients by antiemetic risk.[4, 24] Within each stratum of risk, we calculated the median number of antiemetic interventions. Finally, the presence of spatial autocorrelation was tested by calculating Moran’s I statistic for the residuals of the multivariate model. |
| Results | | | | |
| Participants | 13* | (a) Report numbers of individuals at each stage of study—eg numbers potentially eligible, examined for eligibility, confirmed eligible, included in the study, completing follow-up, and analysed | Results | 51,809 anesthetic case records of patients undergoing surgery at the University of Utah were extracted from the Utah MPOG 2021 dataset. After excluding cases with missing data, a final dataset of 19,477 cases were analysed in cluster analysis, as detailed in Figure 2, Quorum Flow Chart, with missing data noted therein. Table 1 lists detailed characteristics of the original cohort as well as the final analysis cohort after applying our exclusions criteria |
|  |  | (b) Give reasons for non-participation at each stage | Results | Following the MPOG/ASPIRE quality metric PONV05 Inclusion Criteria,[29] and utilizing information obtained from the Utah local Epic and MPOG instances, we excluded cases with American Society of Anesthesiologists Physical Classification Status (ASA) of 5 or 6, age<18 years, ICU transfers, and cases performed without general anesthesia (including Obstetric, ECT electroconvulsive therapy, and bronchoscopy procedures). Any incomplete address information was excluded. After excluding cases with missing data, a final dataset of 19,477 cases were analysed in cluster analysis, as detailed in Figure 2, Quorum Flow Chart, with missing data noted therein. |
|  |  | (c) Consider use of a flow diagram | Results | We present in Figure 2, a Quorum Flow Chart, with missing data noted therein. After excluding cases with missing data, a final dataset of 19,477 cases were analysed in cluster analysis, as detailed in Figure 2, Quorum Flow Chart, with missing data noted therein. |
| Descriptive data | 14* | (a) Give characteristics of study participants (eg demographic, clinical, social) and information on exposures and potential confounders | Results | The median age in the final cohort was 48 years (IQR 34-63); more than 95% of patients underwent a general anesthetic, often lasting longer than 1 hour (40%); the administration of opioid medication was almost always part of the anesthetic plan (98.5%). ASA class was mostly 1-3 (12%, 47% and 37%, respectively). Approximately one-tenth of patients reported a history of PONV. |
|  |  | (b) Indicate number of participants with missing data for each variable of interest | Results | Any incomplete address information was excluded. After excluding cases with missing data, a final dataset of 19,477 cases were analysed in cluster analysis, as detailed in Figure 2, Quorum Flow Chart, with missing data noted therein. We present in Figure 2, a Quorum Flow Chart, with missing data noted therein. |
|  |  | (c) *Cohort study*—Summarise follow-up time (eg, average and total amount) |  |  |
| Outcome data | 15* | *Cohort study*—Report numbers of outcome events or summary measures over time | Results | *In total, we found 2,260 low RAAP cases, 7,358 median RAAP, and 9,589 high RAAP cases in our final dataset. The cluster had a total of 1,934 included participants residing within it, or approximately 9.93% of the study population. The RR of the whole cluster was 1.44 (p<.001), implying that the risk to receive less risk adjusted antiemetic prophylaxis (low RAAP) was 1.44 times higher for patients living within the cluster than for those living in other CBGs along the Wasatch Front.* |
|  |  | *Case-control study—*Report numbers in each exposure category, or summary measures of exposure |  |  |
|  |  | *Cross-sectional study—*Report numbers of outcome events or summary measures |  |  |
| Main results | 16 | (*a*) Give unadjusted estimates and, if applicable, confounder-adjusted estimates and their precision (eg, 95% confidence interval). Make clear which confounders were adjusted for and why they were included | Results | The RR of the whole cluster was 1.44 (p<.001), implying that the risk to receive less risk adjusted antiemetic prophylaxis (low RAAP) was 1.44 times higher for patients living within the cluster than for those living in other CBGs along the Wasatch Front. The CBG level RR within the cluster ranged between 0 to 3.73 (with RR>1 being a high relative risk), meaning the highest risk of low RAAP was 3.73 times higher living in CBGs within versus outside the cluster. After adjusting for all listed covariates, we found that percentage of age greater than 65 years in a CBG cluster became highly significantly associated with a low RAAP cluster, which would represent guidance congruent care as age protects against PONV. |
|  |  | (*b*) Report category boundaries when continuous variables were categorized | Results | We categorized the PONV risk score into six ordinal levels (0-1, 2, 3, 4, 5, 6+), following the PONV05 standard of MPOG.[29] Finally, we assigned the three levels (below median, median, and above median) RAAP contingent on the number of prophylactic antiemetic interventions received: a high RAAP for an above median number, a median RAAP for cases with an intermediate number, and a low RAAP for cases with a below median number of antiemetic prophylactic interventions compared to other cases in the same PONV risk stratum. ND is an average of five US Census indicators, including proportion of the following: non-Hispanic Black, female headed families, households with public assistance or on food stamps, income below federal poverty level, and unemployment, all derived from the United States Census American Community Survey 5-year estimates from 2016-2020. |
|  |  | (*c*) If relevant, consider translating estimates of relative risk into absolute risk for a meaningful time period |  |  |

Continued on next page

| Other analyses | 17 | Report other analyses done—eg analyses of subgroups and interactions, and sensitivity analyses | Results | First, we stratified our patients by antiemetic risk.[4, 24]  We used univariate and multivariate logistic regressions to investigate the characteristics of the CBG in the identified low RAAP cluster.  We tested previously described or suspected social determinants of health as predictors of disparities:[2, 5] percentage of non-White population, percentage of population with education less than high school, percentage of population below poverty rate, percentage of population married, percentage of elderly (> 65years), and percentage of renters. |
| --- | --- | --- | --- | --- |
| Discussion | | | | |
| Key results | 18 | Summarise key results with reference to study objectives | Discussion | We identified one significant cluster (p < .001) of low RAAP cases in Wasatch Front (Figure 3), comprising 101 CBGs. The cluster identified is an area of low RAAP cases with a high neighborhood disadvantage score (reflecting structural neighbourhood features such as female headed families, households with public assistance or on food stamps, income below federal poverty level, and unemployment), and a relatively higher percentage of Hispanic, non-white patients. Our results remained statistically significant in multi-variate analysis, where we demonstrated that non-white received lower RAAP than self-identified white participants. |
| Limitations | 19 | Discuss limitations of the study, taking into account sources of potential bias or imprecision. Discuss both direction and magnitude of any potential bias | Discussion | First, limiting generalizability, we confined the study population to the catchment area of the University of Utah Hospital, limited to one Health system and one group of anesthesiologists. Another limitation, inherent in all geographic epidemiological retrospective studies, is the ecological fallacy;[9, 45] we did not have individual self-identified race for all patients, as our local instance of MPOG and Epic provide inconsistent information on race and ethnicity, thus this is a data quality gap that constrains our interpretation. We acknowledge potential missing data bias, since we were only able to identify 93.1% of patients’ addresses to the level of the street point address, (which is, however, consistent with other geocoding results).[9, 46] |
| Interpretation | 20 | Give a cautious overall interpretation of results considering objectives, limitations, multiplicity of analyses, results from similar studies, and other relevant evidence | Discussion | Only individual patient-level PONV risk factors should be the guiding principle of risk adjusted antiemetic prophylaxis (RAAP);[2, 4, 5, 24] neither patient race and ethnicity, nor neighborhood disadvantage should impede equitable risk-adjusted antiemetic prophylaxis.[2, 18, 24] By identifying geographic clusters of low RAAP, representing populations experiencing disparities in antiemetic prophylaxis after adjusting for risk, we visualized how geographically defined social determinants of health drive healthcare process disparities within anesthesiology.[9] Our results are consistent with these prior findings but examine perioperative process disparities through a geospatial lens with granular geostatistical methods, considering proximity statistically.[9, 21] |
| Generalisability | 21 | Discuss the generalisability (external validity) of the study results | Discussion | First, limiting generalizability, we confined the study population to the catchment area of the University of Utah Hospital, limited to one Health system and one group of anesthesiologists. Furthermore, racial and ethnic diversity of populations vary across the US, somewhat limiting the external validity of our study to states with lower minority representation. Expanding our approach to include areas of economic diversity among the Hispanic or other minoritized populations (e.g. Texas, South Florida, California) would further elucidate this relationship. |
| Other information | |  | | |
| Funding | 22 | Give the source of funding and the role of the funders for the present study and, if applicable, for the original study on which the present article is based | Authors Contributions and Funding | Authors Contributions and Funding |

*Give information separately for cases and controls in case-control studies and, if applicable, for exposed and unexposed groups in cohort and cross-sectional studies.

**Note:** An Explanation and Elaboration article discusses each checklist item and gives methodological background and published examples of transparent reporting. The STROBE checklist is best used in conjunction with this article (freely available on the Web sites of PLoS Medicine at http://www.plosmedicine.org/, Annals of Internal Medicine at http://www.annals.org/, and Epidemiology at http://www.epidem.com/). Information on the STROBE Initiative is available at www.strobe-statement.org.
